# Reducing the risk of fetal distress with sildenafil study (RIDSTRESS): a double-blind randomised control trial

**DOI:** 10.1186/s12967-016-0769-0

**Published:** 2016-01-14

**Authors:** Liam Dunn, Vicki Flenady, Sailesh Kumar

**Affiliations:** Translating Research Into Practice Centre, Mater Research Institute–University of Queensland, Aubigny Place, Raymond Terrace, South Brisbane, QLD 4101 Australia; School of Medicine, The University of Queensland, Brisbane, Australia

**Keywords:** Intrapartum fetal compromise, Fetal distress, Caesarean section, Sildenafil citrate

## Abstract

**Background:**

Labour is perhaps the most hazardous time in pregnancy. As many as 20 % of cerebral palsy cases in term infants result from intrapartum events and up to 63 % of babies who develop intrapartum compromise have no prior risk factors. Sildenafil citrate (SC), a phosphodiesterase 5 inhibitor, improves uterine blood supply through vasodilatation and potentially could improve placental perfusion and hence reduce the risk of intrapartum fetal hypoxia. The aim of this study is to evaluate the efficacy of SC to reduce the risk of intrapartum fetal compromise and the need for emergency operative delivery.

**Methods/design:**

This is a single centre, double-blind, randomised, phase II clinical trial of SC or placebo given during labour to women (18–50 years of age) with a single, appropriately grown, non-anomalous baby at term (37–42 weeks gestation). Those with cardiovascular, renal, hepatic, ocular or hypertensive disease or contraindication to SC will be excluded. Participants will be randomised to either SC 50 mg or placebo capsules eight hourly (SC maximum 150 mg) to commence when admitted to birth suite for management of labour. Within 3 h of the first dose, a repeat ultrasound scan will be performed to measure any changes in uteroplacental and fetal Doppler indices. Labour will continue otherwise in accordance with hospital clinical guidelines. The primary outcome is emergency caesarean section for intrapartum fetal compromise. Secondary outcomes include the effect of SC on fetal and uteroplacental blood flow, meconium liquor, fetal heart rate abnormalities and neonatal outcomes (admission to neonatal intensive care, Apgar <7 at 5 min, cord pH <7.1 or lactate >4.0 mmol/L, neonatal encephalopathy, death).

**Conclusion:**

This is the first reported study evaluating the efficacy of SC on reducing the risk intrapartum fetal compromise.

*Trial Registration:* Australian New Zealand Clinical Trial Registry ACTRN12615000319572

## Background

Labour is perhaps the most hazardous time in pregnancy for the fetus. Uterine contractions are associated with an up to 60 % decline in uterine blood flow [1], and this in turn may lead to fetal decompensation. Up to 63 % of babies who become compromised and suffer oxygen deprivation in labour have no prior risk factors [2] and furthermore events in labour account for as many as 20 % of cases of cerebral palsy in term infants [3]. It is estimated that worldwide, of the 7.6 million deaths of children under 5 years of age, almost 700,000 (9.4 %) are as a consequence of intrapartum related complications [4] and that almost 45 % of stillbirths occurring during labour are usually due to hypoxia [3].

In many pregnancies placental function is sufficient to allow adequate growth of the fetus, but may be not be adequate enough to meet the extra demands required during labour. This potentially predisposes these babies to intrapartum hypoxia clinically manifested by fetal heart rate abnormalities and meconium stained liquor. In extreme cases, these babies can be born acidotic or suffer hypoxic ischaemic encephalopathy. If severe enough, these insults not only result in considerable short term morbidity but also significant long term neurodevelopmental consequences. There is increasing evidence that amongst appropriately grown fetuses at term there exists a cohort of babies with evidence of cerebral redistribution similar to that seen in growth restricted fetuses [5, 6]. Cerebral redistribution is an adaptive response to sub-optimal placental function [2], and occurs prior to obvious deterioration in growth velocity.

Previous work from our group has shown that changes in fetal blood flow detected by ultrasound amongst appropriately grown fetuses were highly predictive of intrapartum fetal compromise (“fetal distress”) in labour, as well as reliably excluding those that were not at risk [5, 6]. We have shown that the ratio of the pulsatility index of the fetal middle cerebral and umbilical arteries, the cerebro-umbilical (CU) ratio, identified fetuses that were at risk of intrapartum fetal compromise. Babies with a CU ratio <10th percentile were six times more likely to be delivered by emergency caesarean section for fetal compromise than those with a CU ratio ≥10th percentile. Furthermore, a CU ratio >90th percentile appeared to be protective for fetal compromise with a negative predictive value of 100 %. Additionally, we have shown that umbilical venous flow was lower in fetuses that developed intrapartum compromise [6]. These results suggest that there exists a population of fetuses at term that, though appropriately grown, still display circulatory evidence of reduced placental function.

Levels of angiogenic factors also appear to be altered in pregnancies with suboptimal placental function. Placental growth factor (PlGF) is a member of the vascular endothelial growth factor (VEGF) family. It is predominantly expressed by the placenta but is also present in lower levels in heart, lung, muscle and adipose tissue [7]. It binds specifically to the VEGFR-1 receptor and whilst PlGF’s precise physiological actions are not clear, it has a pivotal role in placental angiogenesis and induces vasodilation of uterine, myometrial, mesenteric, and subcutaneous arteries [7]. This effect is particularly pronounced in uterine arteries during pregnancy [7], suggesting that PlGF contributes to uterine vascular remodelling during gestation. Maternal levels of PlGF are lower in pregnancies that develop early onset pre-eclampsia and/or fetal growth restriction [8, 9]. The low levels of PlGF in both clinical situations likely reflect the underlying pathophysiology whereby defective trophoblast invasion leads to altered expression of angiogenic-related factors [10, 11]. There are however no data relating to levels of maternal PlGF levels and intrapartum compromise at term.

Having developed a technique for prediction of intrapartum compromise we now wish to translate this into the clinical setting in a pilot randomised controlled trial using the phosphodiesterase 5 inhibitor sildenafil citrate (SC) to reduce the risk of fetal compromise in labour. SC has been investigated as a potential agent to enhance fertility and endometrial thickness for implantation [12–14] however much of the evidence to support the use of SC in pregnancy comes from its use in ex vivo and animal models of severe fetal growth restriction [15, 16] and preeclampsia [17–20]. These have demonstrated SC improves uteroplacental blood flow through its vasodilatory effects on umbilical and uterine arteries, with the downstream effect being an increase in average pup birth weight [15].

In humans, SC appears to be well tolerated [21] and has been shown to improve perinatal outcomes in pregnancies complicated by fetal growth restriction or pre-eclampsia [21–23]. A recent small cohort study of severe intrauterine growth restriction showed a tendency towards more live-born children that survived intact to primary discharge after SC intervention [23]. SC may therefore be a potential therapeutic option to improve uteroplacental blood flow in pregnancies complicated by severe fetal growth restriction. Its efficacy in this regard is currently being assessed in a multi-centre, international study currently underway in the United Kingdom, Australia and New Zealand [24]. The available evidence so far suggests that the use of SC at doses of 20–240 mg/day in the second or third trimester of pregnancy is not associated with significant adverse maternal or fetal outcomes [21, 25–32].

Given that the cardiovascular adaptations in fetuses with growth restriction are similar to that seen in term appropriately grown babies who develop intrapartum fetal compromise; it is reasonable to suppose that SC, via its ability to improve uteroplacental perfusion could potentially reduce the risk of intrapartum compromise in term, appropriately grown babies and the need for emergency delivery. It is this premise that is being investigated in the current study.

## Methods and trial design

### Objectives

#### Primary objective

To determine if SC reduces the rate of emergency caesarean section for intrapartum fetal compromise.

#### Secondary objectives

1. To ascertain the influence of SC on fetal and uteroplacental blood flow.
2. To ascertain if SC is associated with an improvement in neonatal outcomes (admission to neonatal intensive care unit, Apgar score <7 at 5 min, cord pH <7.1 or lactate >4 mmol/L, neonatal encephalopathy, death).
3. To ascertain if SC is associated with a reduction in intrapartum fetal heart rate abnormalities.

### Trial design

This is a single site, double-blind, randomised placebo controlled phase II clinical trial to assess the effect of SC on the incidence of intrapartum fetal compromise and emergency caesarean section rates.

Once recruited to this study, participants will have an ultrasound scan performed. Fetal biometry, amniotic fluid index, fetal middle cerebral artery and umbilical artery pulsatility indices will be measured. A 10 mL maternal blood sample will be obtained to measure PlGF levels.

Randomisation will be determined using the *STATA 13* (StataCorp, College Station, Texas) program and will be done by a member of the data management and analysis team at Mater Research Institute. This will ensure the research team is blinded to the allocation of SC and placebo. Participants will be randomised to either the SC or placebo arm in equal numbers (1:1) in block sizes of four and six to yield the appropriate sample size.

A sequentially numbered trial pack will be allocated to each participant. Each trial pack will be prepared by the study site’s pharmacy in accordance with the randomisation schedule. Each trial pack will contain three capsules of either SC or placebo, along with instructions for the attending clinical staff.

The first dose of SC or placebo will be administered by the attending midwife after the participant has been transferred to the birth suite for management of labour. Labour is defined as cervical dilatation ≥4 cm with uterine contractions or at commencement of induction of labour with artificial rupture of membranes and/or oxytocin infusion.

Following the first dose of SC or placebo, a repeat ultrasound scan will be performed within 3 h to measure the previously described maternal and fetal vascular Doppler parameters. The purpose of the second ultrasound scan is to ascertain changes, if any, in fetal blood flow as a result of the drug (i.e. SC or placebo).

Clinicians managing the pregnancy will be blinded to the results of the ultrasound scans and the randomisation schedule to ensure labour is managed according to normal institutional protocols and guidelines. This will ensure obstetric care is consistent across both arms of the study.

The following intrapartum outcomes will be recorded: mode of delivery, indication for operative delivery, duration of labour, fetal heart rate abnormalities (in accordance with the National Institute for Health and Care Excellence cardiotocograph categorisation [33]), intrapartum and postpartum haemorrhage, meconium stained liquor, need for fetal blood sampling as well as other any serious intrapartum event (cord prolapse, abruption and maternal collapse). Neonatal outcomes will also be recorded—birth weight, gender of baby, Apgar scores, cord arterial pH and lactate levels, need for resuscitation, indication for and duration of neonatal intensive care unit admission, seizures, neonatal encephalopathy and death.

### Study population

#### Inclusion criteria

- Women between 18–50 years who are able to give informed consent.
- Singleton pregnancy between 37 + 0–42 + 0 weeks gestation.
- Cephalic presentation.
- Appropriately grown (>2500 g) [34] fetus without any known structural, chromosomal or genetic abnormality.
- Admitted in early labour (cervical dilatation <4 cm) or for scheduled induction of labour.
- Planning a vaginal delivery.

#### Exclusion criteria

- Women <18 years old and those unable to give informed consent.
- Women with pre-existing cardiac disorders, stroke, hypotension or hypertension, retinitis pigmentosa, kidney or liver abnormalities, sickle cell anaemia, stomach ulcers or any other bleeding disorders.
- Women taking any anti-hypertensive medication, alpha-adrenergic blocking agents, calcium channel blockers (verapamil), amyl nitrate, nicorandil, nitrates (including glyceryl trinitrate or isosorbide salts), sodium nitroprusside, bosentan, fosamprenavir and ritonavir combination, hepatic enzyme inhibitors CYP3A4 (including itraconazole, ketoconazole, ritonavir, cimetidine, erythromycin, saquinavir, darunavir), hepatic enzyme substrates (CYP3A4 and beta-adrenergic blocking agents), medications used to treat pulmonary arterial hypertension, and other phosphodiesterase type 5 inhibitors.

### Sample size calculation

The proposed sample size is based on our previous pilot data which showed that the incidence of emergency caesarean section for fetal compromise in the cohort of fetuses with a CU ratio <10th centile was approximately 36 % [5]. If SC is successful in halving the rate of caesarean section in this cohort to 18 %, a total sample size of 910 women (80 % power, alpha of 0.05) would be required. Assuming a 10 % drop out rate, a sample of 1012 women should be sufficient to adequately address the research objectives.

Analysis of this study will be on an intention to treat (ITT) basis. Based on current birthing rates at the trial site the proposed number of participants required is pragmatic and should be easily achievable within the study period of 3 years.

An interim analysis will be performed after the first 100 patients have been recruited. Ongoing safety analyses will be performed after each subsequent 100 participants.

In this study, each participant’s records will be reviewed within 48 h of delivery, for collection of outcome data. This will also allow rapid identification of any immediate adverse events. Additionally, there will be a follow up phone call within 2 weeks of discharge from hospital to ascertain if there any issues relating to the study that require additional follow up. All follow up and data collection will be conducted by a two members of the investigating team. Up to date reports on safety will be provided to the study sponsor and local ethics committee upon request.

### Study review and approval

The protocol, patient information and consent form and other accompanying material that will be provided to participants have been reviewed and approved by the Mater Human Research Ethics Committee (Reference No: HREC EC00332).

## Discussion

The aim of this study is to evaluate if SC given during labour can reduce the risk of an emergency caesarean section for fetal compromise. Additionally, intrapartum outcomes including mode of delivery, fetal heart rate abnormalities and meconium liquor and neonatal outcomes including nursery admission, Apgar score, cord blood acidaemia and hypoxic ischaemic encephalopathy will be evaluated.

Intrapartum hypoxia contributes a significant burden to maternal and neonatal morbidity. Despite many advances in antenatal detection, intrapartum management and neonatal care, the majority of cases of intrapartum fetal compromise occur in otherwise ‘low risk’ women. To our knowledge, this is the first study designed to measure the efficacy of intrapartum SC in reducing emergency caesarean sections for fetal compromise. The population being investigated is ‘low risk’ such that the outcomes from this study will have broad generalisability and the study is powered to measure a significant reduction of 50 % in the rate of emergency caesarean sections amongst women whose baby has a CU ratio <10th centile. As this study is being conducted in a large tertiary obstetric referral centre with over 10,000 deliveries annually the recruitment target is anticipated to be achievable.

There currently is no therapeutic agent to reduce the risk of intrapartum fetal compromise, and emergency delivery, often by caesarean section remains the only course of action if vaginal delivery is not immediately achievable. Emergency caesarean sections are associated with poorer neonatal outcomes [35]. This is likely a reflection on the indication for such urgent delivery. Any reduction of the risk of requiring an emergency caesarean section is key to improving both perinatal and maternal health outcomes globally. Furthermore, there is currently much emphasis on reducing the overall rates of caesarean section. The World Health Organisation (WHO) recommends a target of 15 % [36], a rate beyond which no additional reductions in maternal, neonatal or infant mortality have been observed [37]. Increasing maternal age and increased prevalence of obesity may in part contribute to increased caesarean rates [38–40] however convenience, patient autonomy and health care resources and system structure also influence higher caesarean rates [40]. The rationale for the target caesarean rate is based on limiting maternal and neonatal morbidity and mortality related to caesarean section. Current global caesarean section rates are far in excess of the WHO target rate, exceeding 25 % in the United States [41–43], the United Kingdom [44] and Australia [45]. Whilst no one singular effective reduction strategy exists, reducing the incidence of fetal compromise would not only reduce adverse neonatal outcomes, but also help reduce overall caesarean section rates.

There is a good rationale for the use of SC to improve uteroplacental blood flow and hence, fetal oxygenation. It is a well-tolerated agent with no known teratogenic or toxic effects at doses used in this study. Additionally it would be given for a relatively short duration, i.e. only during labour. In resource poor settings, fetal monitoring, surgical expertise and neonatal care are often inadequate to provide optimal perinatal care for both mother and baby. A cost effective and well tolerated pharmacological agent with a long shelf life, like SC, could potentially address this important clinical problem and dramatically improve perinatal outcomes. Its implementation however could be applicable and achievable in any resource setting and have a far reaching impact on obstetric care.

## Conclusion

Perinatal morbidity and mortality associated with intrapartum fetal hypoxia persists as a global health challenge. There is currently no treatment for this other than urgent operative delivery, facilities for which are not always immediately available in low and middle income countries. The *RIDSTRESS* study will test the efficacy of SC to reduce the need for emergency caesarean section when intrapartum fetal hypoxia is suspected. If SC is demonstrated to confer a therapeutic benefit, we believe that it could provide a safe and affordable adjunct to intrapartum management in all resource settings.
